# Publisher Correction: Exciton transport in molecular organic semiconductors boosted by transient quantum delocalization

**DOI:** 10.1038/s41467-024-47500-4

**Published:** 2024-04-11

**Authors:** Samuele Giannini, Wei-Tao Peng, Lorenzo Cupellini, Daniele Padula, Antoine Carof, Jochen Blumberger

**Affiliations:** 1 Department of Physics and Astronomy and Thomas Young Centre, University College London, WC1E 6BT London, UK; 2 Dipartimento di Chimica e Chimica Industriale, Universitá di Pisa, Via G. Moruzzi 13, 56124 Pisa, Italy; 3 Dipartimento di Biotecnologie, Chimica e Farmacia, Universitá di Siena, Via A. Moro 2, 53100 Siena, Italy; 4 Laboratoire de Physique et Chimie Théoriques, CNRS, UMR No. 7019, Université de Lorraine, BP 239, 54506 Vandoeuvre-lés-Nancy Cedex, France; 5 Present Address: Laboratory for Chemistry of Novel Materials, University of Mons, Place du Parc 20, 7000 Mons, Belgium

**Keywords:** Theory and computation, Nanoscale materials

Correction to: *Nature Communications* 10.1038/s41467-022-30308-5, published online 19 May 2022

The original version of this Article contained an error in Table 2. The data entries for the a6T b-direction (4.5 ± 0.3 and 40 ± 2) and the PDI b-direction (9 ± 3) have been misplaced and should be shifted one column to the right. This has been corrected in both the PDF and HTML versions of the Article.

