# Supplementary material for: Exciton transport in molecular organic semiconductors boosted by transient quantum delocalization
Source: Nat Commun. 2022 May 19;13:2755. doi: 10.1038/s41467-022-30308-5 (PMC9120088; doi:10.1038/s41467-022-30308-5)
Supplement: Supplementary file 1 — Supplementary Information [file 41467_2022_30308_MOESM1_ESM.pdf]

Supplementary Information: Exciton transport in molecular organic semiconductors boosted by transient quantum delocalization

Samuele Giannini[1, †,*], Wei-Tao Peng[1], Lorenzo Cupellini[2], Daniele Padula[3], Antoine Carof[4], and Jochen Blumberger[1,*]

[1]Department of Physics and Astronomy and Thomas Young Centre, University College London, WC1E 6BT London, UK.

[†]Current address: Laboratory for Chemistry of Novel Materials, University of Mons, Place du Parc 20, 7000 Mons, Belgium

[2]Dipartimento di Chimica e Chimica Industriale, Universitá di Pisa, Via G. Moruzzi 13, 56124 Pisa, Italy

[3]Dipartimento di Biotecnologie, Chimica e Farmacia, Universitá di Siena, Via A. Moro 2, 53100 Siena, Italy.

[4]Laboratoire de Physique et Chimie Théoriques, CNRS, UMR No. 7019, Université de Lorraine, BP 239, 54506 Vandoeuvre-lés-Nancy Cedex, France.

[*]Corresponding Authors, e-mail: samuele.giannini.16@ucl.ac.uk, j.blumberger@ucl.ac.uk

Supplementary Note 1

Excitation energies

The vertical excitation energies as well as the adiabatic excitation energies for the lowest singlet excited state (S1) of ANT, a6T, PDI, DCVSN5 and Y6 molecules are reported in Supplementary Table 1. These data refer to optimized geometries of the single molecules in gas-phase.

Supplementary Table 1: Excitation (eV) for the lowest-energy (singlet) state of ANT, a6T, PDI, DCVSN5 and Y6.[a]

	Functional	S1 vert.	f [c]	S1 adiab.	S1 Exp. [d]
ANT	CAM-B3LYP	3.70	0.086	3.41	3.3-3.4
	ωB97X-D	3.71	0.089	3.43	
a6T[b]	CAM-B3LYP	2.96	2.042	2.68	2.5
	ωB97X-D	3.03	2.060	2.74	
PDI	CAM-B3LYP	2.85	0.752	2.65	2.37
	ωB97X-D	2.89	0.758	2.68	
DCVSN5	CAM-B3LYP	2.79	2.049	2.64	2.13
	ωB97X-D	2.86	2.061	2.69	
Y6	CAM-B3LYP	2.35	2.421	2.23	1.70
	ωB97X-D	2.45	2.436	2.32	

[a] Basis set is fixed to 6-31g(d,p) as commonly used in the literature for similar systems[1–4]. [b] The geometry for a6T was kept coplanar as done in Ref.[2] since oligothiophenes tend to crystallize in the solid state with coplanar thiophene units[2]. [c] The calculated oscillator strengths are reported in a.u. [d] Experimental values are taken for solution and gas-phase molecules from Ref.[5,6] in the case of ANT, Ref.[7,8] for a6T, Ref.[9] for a similar PDI derivative, Ref.[10] for DCVSN5 and Ref.[11] for Y6.

The first important observation is the fact the two long-range corrected functionals give very similar values both for energies and oscillator strengths (f) of the lowest-energy excitations of the different systems, pointing to a robust description of the transition density of these excitations by different functionals. As the following calculations are aimed at estimating correctly electronic couplings, as explained in the main text, a good evaluation of the transition densities is the most important issue. It is also worth noting that the second lowest (singlet) excited state as found from both functionals is about 0.5-1.0 eV above S1. This means that the Frenkel approximation describing the excited state of the system as a combination of locally excited S1 states is a good first approximation to study transport properties in these OSs.

We also plot the Natural Transition Orbitals (NTOs)[12] corresponding to the S1 excited state in Figure 1 of the main text for all the investigated molecules. The NTOs offer a useful way of visualizing which orbitals give the largest contribution to a given single-particle excitation (the NTOs are obtained here by using the NTOBuilder tool[13]). For all systems we can see that the NTOs with the largest contributions

are indeed essentially the same as the HOMO and LUMO orbitals of the single molecules.

Supplementary Note 2

Internal exciton reorganization energies

We have performed (internal) exciton reorganization energy calculations (Eq. 10 in the main text) with three well-established long-range corrected hybrid functionals: CAM-B3LYP, ωB97X-D and M06-2X. Results are shown in Supplementary Tables 2 and 3 and compared with literature data. It is clear that exciton reorganization energies are quite robust with choice of the functional and they change very little with increasing basis-set size. They also agree well with literature data.

Supplementary Table 2: Reorganization energy functional dependence. All values are in meV (basis-set used: 6-31G(d,p)).

Systems	CAM-B3LYP	ωB97X-D	M06-2X	Lit.
ANT	572	561	588	530^a, 589^b
a6T	558	562	554	-
PDI	390	405	391	-
DCVSN5	294	320	278	328^b
Y6	250	267	225	242^c, 197^d

[a] Taken from Ref.[14], level of theory: SCS-CC/cc-pVTZ [b] Taken from Ref.[15], reorganization energy from normal mode analysis, level of theory: ωB97X-D/6-31G(d,p) [c] Taken from Ref.[16], level of theory: M06-2X/6-311g(d,p) [d] Taken from Ref[17], level of theory: optimally-tuned range separated hybrid with dielectric screening.

Supplementary Table 3: Reorganization energy basis-set dependence . All values are in meV (functional used: CAM-B3LYP).

Systems	6-31G(d)	6-31G(d,p)	6-311G(d,p)
ANT	572	572	596
a6T	559	558	572
PDI	390	390	405
DCVSN5	295	294	302
Y6	247	250	251

Supplementary Method 1

Calculation of excitonic couplings

We analyse here various flavours of the excitonic coupling approaches as defined in the main text. The electronic coupling is one of the main ingredients of the Frenkel exciton Hamiltonian employed in this work. We evaluated excitonic couplings using the full Coulomb integral, V_{Coulomb}, in Eq. 11 main text, the TrESP approach, V_{TrESP}, in Eq. 12 main text and the point-dipole approximation (PDA), V_{PDA}, in Eq. 8 below, for all the systems considered here. These values have been compared with the total excitonic coupling, V, obtained using the multi-state fragment excitation difference fragment charge difference approach (MS-FED-FCD) described below. The objective is to assess the extent of the short-range coupling in these closely packed OS solids and the different levels of approximation of the long-range Coulomb interaction. We note in passing that, to check the quality of the TrESP charges found from the ESP fitting procedure (as described in the main text), we have made sure that the sum of the TrESP charges is zero (as it would be the case if the full transition density were integrated over all space) and also that the dipole moments obtained directly from the charge densities, $\mu = \int d\mathbf{r}_I \rho^T(\mathbf{r}_I)\mathbf{r}_I$, are identical with those calculated from the atomic TrESP charges, $\mu = \sum_I q_I \mathbf{r}_I$.

For the calculation of MS-FED-FCD couplings (V) and Coulomb contributions (V_{Coulomb}) as well as for fitting the TrESP charges, we have used CAM-B3LYP[18] functional for a6T, PTCDI-H and Y6 (as suggested in Ref.[4,19]) and ωB97X-D[20] for ANT and DCVSN5 (for a more consistent comparison with Ref[1]). The results are reported in Table 1 of the main text for three of the closest crystal pairs of the investigated OSs. As reported in the main text, we observe that V_{Coulomb} is generally very close to the total coupling V. The mean relative unsigned error (MRUE) is about 0.7% for the crystal structures (and about 7% along MD). This small discrepancy can be attributed to the missing short-range part, V_{Short}. The observation that $V \approx V_{\mathrm{Coulomb}}$ lays the groundwork for efficient approximations of the long-range Coulomb interaction using TrESP charges (see below).

Notably, we also found that the two long-range corrected functionals give very similar values for the Coulomb couplings of the analysed systems. This is because the transition densities are described similarly by both functionals.

Multi-state FED-FCD

In this section we present two useful diabatization schemes: the fragment charge difference (FCD)[21,22] and the fragment excitation difference (FED)[23] methods, and their combination in the general multi-state FED-FCD (MS-FED-FCD) scheme,[24–26] used in this work to obtain total excitonic couplings (V in the main text) between maximally localized Frenkel exciton states, that can be used as a benchmark for

more approximate methods as discussed in the main text.

We start by considering the case of a dimer composed of two molecules donor (k) and acceptor (l). The FED scheme can recover the diabatic (localized) basis from delocalized excited states by using an additional operator Δx, which measures the difference in excitation number between the donor (k) and acceptor (l) molecules. The elements of the Δx matrix are given in terms of "excitation densities", defined as the sum of attachment (electron) and detachment (hole) densities[27]:

$$\Delta x_{ij} = \int_{\mathbf{r}\in k} \rho_{ij}^{\mathrm{ex}}(\mathbf{r})\mathrm{d}\mathbf{r} - \int_{\mathbf{r}\in l} \rho_{ij}^{\mathrm{ex}}(\mathbf{r})\mathrm{d}\mathbf{r} \tag{1}$$

where i and j are two adiabatic states and ρ_{ij}^{ex} is the excitation density, defined as,

$$\rho_{ij}^{\mathrm{ex}}(\mathbf{r}) = \rho_{ij}^{\mathrm{Att}}(\mathbf{r}) + \rho_{ij}^{\mathrm{Det}}(\mathbf{r}) \tag{2}$$

The quantity Δx has its extrema when the excitation is entirely localized on either the donor (k) or acceptor (l). Without loss of generality, assuming that the adiabatic states i and j are the combination of two diabatic states, $|k^*l\rangle$ localized on k, and $|kl^*\rangle$ localized on l, the eigenvectors of the 2×2 Δx matrix represent the transformation from the adiabatic to the diabatic basis ($\mathbb{U}$), and the eigenvalues are either 1 or -1 for $|k^*l\rangle$ and $|kl^*\rangle$[23,24,28]. The diagonal matrix of adiabatic energies ($\mathbb{H}^{\mathrm{ad}}$) can be transformed into the diabatic basis of $|k^*l\rangle$ and $|kl^*\rangle$ by $\mathbb{U}^{\dagger}\mathbb{H}^{\mathrm{ad}}\mathbb{U} = \mathbb{H}$. The electronic coupling between these states is found as the off-diagonal element of $\mathbb{H}$.

The same strategy can be applied to computing the coupling to charge transfer (CT) states. The FCD method uses an additional operator Δq to localize the electronic charge:[21]

$$2\Delta q_{ij} = \int_{\mathbf{r}\in k} \rho_{ij}(\mathbf{r})\mathrm{d}\mathbf{r} - \int_{\mathbf{r}\in l} \rho_{ij}(\mathbf{r})\mathrm{d}\mathbf{r} \tag{3}$$

where $\rho_{ij}(r)$ is the transition density between states j and i, if $j \neq i$, and the state density if $j = i$. Similarly to the FED case, the matrix Δq can be diagonalized to obtain locally excited states (such as kl^* and k^*l) with eigenvalue 0, and CT states, such as k^-l^+ or k^+l^-, with eigenvalues 1 or -1, respectively. This is similar to the FED when the adiabatic states are combination of only two diabatic states. However, in the systems analysed in this work a 2 state diabatization procedure is not sufficient to retrieve excitonic couplings between completely de-mixed and localized (Frenkel) exciton states, which in our case, form the state space for the Frenkel Hamiltonian in Eq. 2 main text.

To overcome this limitation we employed a multi-state FED-FCD procedure. This approach is a generalization of the FED and FCD methods just described[22,24,28] and can make use of multiple adiabatic eigenstates of the donor-acceptor supermolecular system to recover maximally localized and decoupled diabatic states. This strategy was previously successfully employed to recover excitonic couplings in light-

harvesting and biological systems[24,25] as well as bridged donor-acceptor moieties[28]. For a more detailed description of this approach we refer to Ref.[22,24]. In brief this algorithm starts from the definitions of the additional operators Δx and Δq defined before. For CT states such as k^-l^+ and k^+l^- Δx should be zero, while $\Delta q = \pm 1$ (albeit small numerical uncertainties[22]). On the contrary, for localized Frenkel-Exciton (XT) states such as kl^* and k^*l, Δx equals $+1$ and -1, respectively, and $\Delta q = 0$. We can define the matrix

$$\mathbb{D} = (\Delta q)^2 - (\Delta x)^2 \tag{4}$$

which then has eigenvalues +1 and -1, respectively for subspaces of CT and XT states. The first step of the multi-FCD-FED scheme is to diagonalize $\mathbb{D}$ to separate CT and XT subspaces, similarly to multi-state FCD (previously developed in Ref.[22]) :

$$\mathbb{U}_1 \mathbb{D} \mathbb{U}_1^\dagger = \mathbb{D}' \tag{5}$$

The same transformation $\mathbb{U}_1$ is applied to Δx and Δq, to obtain $\Delta x'$ and $\Delta q'$. Now, the states can be assigned as either CT or XT depending on the value of $\mathbb{D}'$. $\Delta x'$ and $\Delta q'$ can be each diagonalized within the XT and CT subspaces respectively, so that:

$$\begin{aligned} \mathbb{U}_2 \Delta x' \mathbb{U}_2^\dagger &= \Delta x'' \\ \mathbb{U}_2 \Delta q' \mathbb{U}_2^\dagger &= \Delta q'' \end{aligned} \tag{6}$$

where $\Delta x''$ is diagonal only in the XT block, whereas $\Delta q''$ is diagonal only in the CT block. $\mathbb{U}_2$ is block diagonal and rotates the XT and CT subspaces separately. Within the XT subspace, the diagonal values of $\Delta x''$ can be used to separate kl^* (XT1) states from k^*l (XT2) states. At the same time, $\Delta q''$ can be used to divide CT states in k^-l^+ (CT1) and k^+l^- (CT2). At this point, the four subspaces have been separated. The diagonal electronic Hamiltonian $\mathbb{E}$ can be transformed to this basis as $\mathbb{U}_2 \mathbb{U}_1 \mathbb{E} \mathbb{U}_1^\dagger \mathbb{U}_2^\dagger = \mathbb{H}''$. Finally, we require that the Hamiltonian of each subspace is diagonal, *i.e.* that the states of the same subspace are not coupled to each other.[22]

$$\mathbb{U}_3 \begin{pmatrix} \mathbb{H}''_{\text{CT1}} & \mathbb{H}''_{\text{CT1,CT2}} & \mathbb{H}''_{\text{CT1,XT1}} & \mathbb{H}''_{\text{CT1,XT2}} \\ \mathbb{H}''_{\text{CT2,CT1}} & \mathbb{H}''_{\text{CT2}} & \mathbb{H}''_{\text{CT2,XT1}} & \mathbb{H}''_{\text{CT2,XT2}} \\ \mathbb{H}''_{\text{XT1,CT1}} & \mathbb{H}''_{\text{XT1,CT2}} & \mathbb{H}''_{\text{XT1}} & \mathbb{H}''_{\text{XT1,XT2}} \\ \mathbb{H}''_{\text{XT2,CT1}} & \mathbb{H}''_{\text{LH2,CT2}} & \mathbb{H}''_{\text{XT2,XT1}} & \mathbb{H}''_{\text{XT2}} \end{pmatrix} \mathbb{U}_3^\dagger = \begin{pmatrix} \epsilon'''_{\text{CT1}} & \mathbb{H}'''_{\text{CT1,CT2}} & \mathbb{H}'''_{\text{CT1,XT1}} & \mathbb{H}'''_{\text{CT1,XT2}} \\ \mathbb{H}'''_{\text{CT2,CT1}} & \epsilon'''_{\text{CT2}} & \mathbb{H}'''_{\text{CT2,XT1}} & \mathbb{H}'''_{\text{CT2,XT2}} \\ \mathbb{H}'''_{\text{XT1,CT1}} & \mathbb{H}'''_{\text{XT1,CT2}} & \epsilon'''_{\text{XT1}} & \mathbb{H}'''_{\text{XT1,XT2}} \\ \mathbb{H}'''_{\text{XT2,CT1}} & \mathbb{H}'''_{\text{LH2,CT2}} & \mathbb{H}'''_{\text{XT2,XT1}} & \epsilon'''_{\text{XT2}} \end{pmatrix} \tag{7}$$

The unitary matrix $\mathbb{U}_3$ is block diagonal, and each block of $\mathbb{U}_3$ diagonalizes each of the CT1, CT2, XT1, and XT2 blocks of the Hamiltonian. The final Hamiltonian $\mathbb{H}'''$ in Eq. 7 contains the diabatic energies ϵ on the diagonal, and the electronic couplings in the off-diagonal blocks.

Point dipole approximation

As it is customary in (dipole-based) Förster theory, at sufficiently large distances between sites (i.e. distance larger than the dimension of the interacting molecules), the transition densities of donor and acceptor can be approximated with the first non-zero term in a multipole expansion, i.e. the transition dipole moment. This approximation is referred to as point dipole approximation (PDA) and the related excitonic coupling, V_{PDA}, can be written as,

$$V_{kl}^{\mathrm{PDA}} = \frac{\mu_k \cdot \mu_l}{r_{kl}^3} - \frac{3(\mu_k \cdot \mathbf{r}_{kl})(\mu_l \cdot \mathbf{r}_{kl})}{r_{kl}^5} \tag{8}$$

where $\mathbf{r}_{kl}$ is the vector distance between k and l molecules and μ_k and μ_l their respective transition dipole moments. We found that the PDA approximation breaks down for all molecules except ANT and should not be used (see Supplementary Note 3).

Supplementary Note 3

Excitonic couplings

The importance of including multiple states in MS-FED-FCD diabatization

It is important to note that in many cases, and also for the systems investigated in this work, a 2 state adiabatic basis, employed in the diabatization procedure to calculate the reference coupling V, is not sufficient to retrieve the coupling between completely localized states. In fact, often the adiabatic states are linear combination of a number of diabatic states of both donor and acceptor. Moreover, charge-transfer states can mix with Frenkel exciton states, and vice-versa[22,28]. To overcome this difficulty and recover the coupling between completely de-mixed and localized (Frenkel) exciton states –which form the state space for the Frenkel Hamiltonian in Eq. 2 – we employed the aforementioned multi-state diabatization (MS-FED-FCD) (see Supplementary Method 1).

The MS-FED-FCD diabatization procedure was carried out using 20 excited states of the supermolecular donor-acceptor system to ensure a complete de-mixing between excitations of different nature (e.g. charge transfer and other excitonic states) and an optimal reconstruction of localized Frenkel exciton states and related couplings. See convergence of V with the number of states in Supplementary Figure 1 for the DCVSN5 system. At least 10 of the lower lying excited states are necessary to reach a converged value for V.[29]

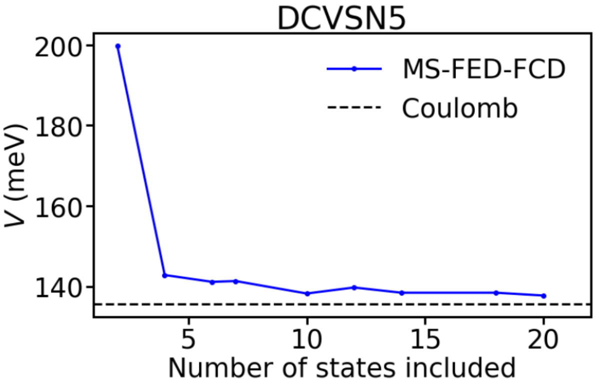

Supplementary Figure 1: Convergence of V coupling as a function of the number of adiabatic states included in the MS-FED-FCD diabatization. Dashed black line indicates the V_{Coulomb} towards which V is converging pointing to a negligibly small short range contribution.

As a complementary analysis to Figure 2 in the main text, where there is an extremely good agreement between V obtained using a converged number of states in the diabatization procedure and both V_{Coulomb} and V_{TrESP}, in Supplementary Figure 2 we report the same correlation but using only 2 states in the MS-FED-FCD (i.e. the regular FED). It is clear that the correlation is no longer optimal and the total excitonic

coupling V between Frenkel exciton states includes unwanted mixing with excited states of different nature that yield, in most cases, an overestimated coupling value.

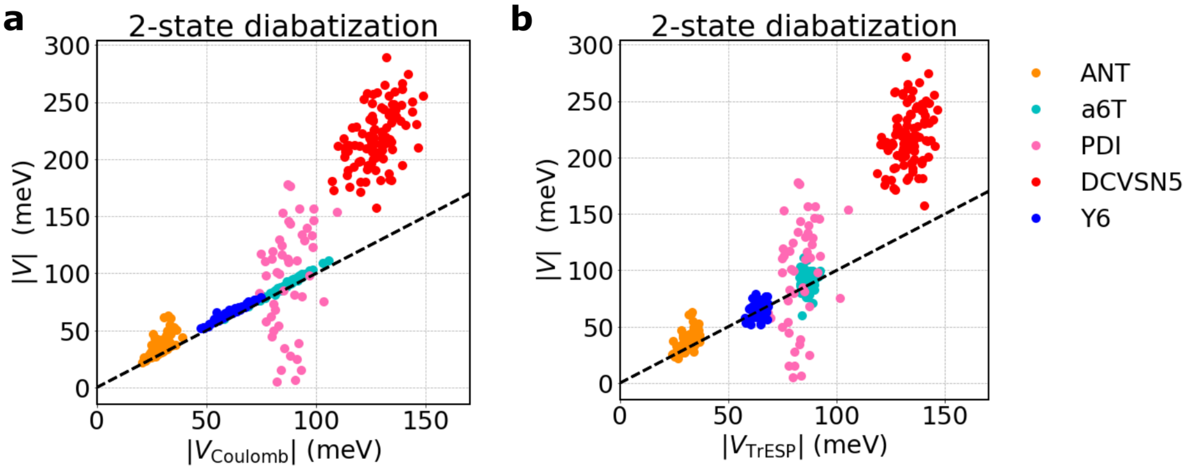

Supplementary Figure 2: Two state diabatization. Correlation between the total excitonic coupling V, found using MS-FED-FCD with only 2 adiabatic states included in the diabatization procedure, and (**a**) V_{Coulomb} and (**b**) V_{TrESP} obtained for P_b, of ANT and a6T and P_a, P_{a1}, P_{a2} of PDI, DCVSN5 and Y6 respectively, extracted from MD trajectories of the various systems.

Excitonic coupling fluctuations

V_{TrESP} obtained by using the ESP fitting procedure described in the *Method* section is in very good agreement with V_{Coulomb} for all systems and crystal pairs investigated (see Table 1 in the main text). The notable difference is the fact that TrESP couplings are readily calculated without the need of repeating an electronic structure calculation for each different geometry. This constitutes a great advantage of using TrESP approach in combination with FE-SH, as it permits calculating many thousands of coupling elements (and related nuclear gradients) at each step along the MD, thus allowing the study of large nano-scale systems. Additionally, this approach allows an efficient calculation of both couplings and coupling fluctuations beyond nearest neighbour pairs and to account for long-range interactions within the (Frenkel) Hamiltonian of these systems. These interactions are of vital importance to get a reliable estimate of the diffusion constant as it was recently pointed out in Ref.[30] and Ref. [31]. The TrESP approach (combined with FE-SH) can help going beyond model Hamiltonians where only fixed nearest neighbour excitonic couplings are considered[1,32].

During the FE-SH simulation we keep the TrESP charges frozen to the values obtained in the parametrization step (see below). Thus, the time dependence of the TrESP excitonic couplings is due to the thermal fluctuations of the nuclei only. Fluctuations in TrESP excitonic couplings due to potential changes in the transition densities and thus transition charges are neglected. To assess to which extent this is a good approximation and how well the TrESP approach can capture the fluctuations of the excitonic couplings,

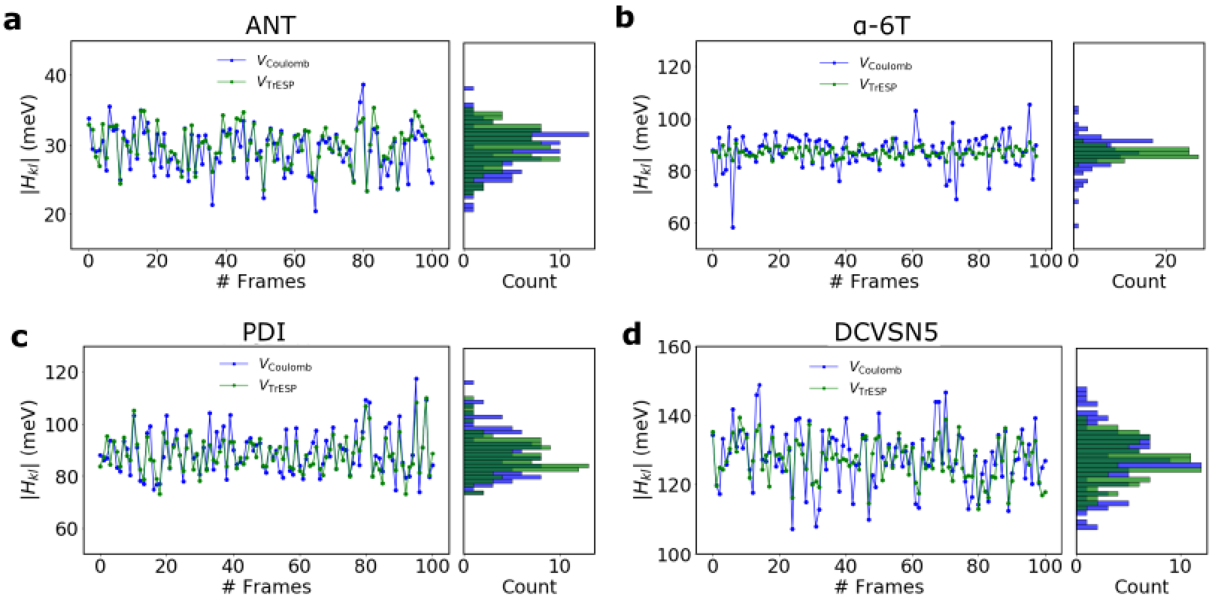

Supplementary Figure 3: Excitonic coupling fluctuations for the investigated OSs. The couplings are evaluated (for P_b of ANT and a6T and P_a, P_{a1} of PDI and DCVSN5, respectively) with V_{TrESP} and V_{Coulomb} for a series of about 100 MD snapshots. The distribution of these couplings are shown as well.

we computed the Coulomb couplings with updated transition densities at each MD step and compared them to the TrESP excitonic couplings with frozen transition charges along 100 snapshots taken from an MD simulation (excitonic couplings for crystal pairs P_b, of ANT and a6T and P_a, P_{a1} of PDI, DCVSN5, respectively). To obtain optimal TrESP charges that partially account for more "exotic" configurations during the parametrization, in this case, we carried out the ESP fitting procedure for 50 different structures along MD simulations for each system and then averaged the TrESP charges obtained over all structures (instead of using just a single molecular structure). A sign-tracking procedure was adopted to keep consistent the sign of the charges when doing the average of TrESP charges. The results are reported in Supplementary Figure 3 along with related coupling distributions.

We can observe that for all the systems the TrESP approach is able to capture the mean value of the coupling extremely well. Interestingly, the coupling fluctuations and the structural dependence of the excitonic couplings are reasonably well captured for ANT, PDI and DCVSN5 as well. In a6T, the TrESP coupling fluctuations are somewhat underestimated compared to the V_{Coulomb}. The explanation for this behaviour is simply that for the first three molecules (ANT, PDI and DCVSN5) the rigidity of the aromatic structure and extended conjugation ensure that the transition density does not change significantly along the dynamics. Thus, fixed TrESP charges provide a very good approximation for these crystals. On the contrary, having a more flexible structure, a6T shows larger structural variations along MD. A similar conclusion holds for Y6 as well. For these cases, fixed transition charges do not capture as well the dependence of the transition density on the geometry. However, excitonic coupling fluctuations (as found

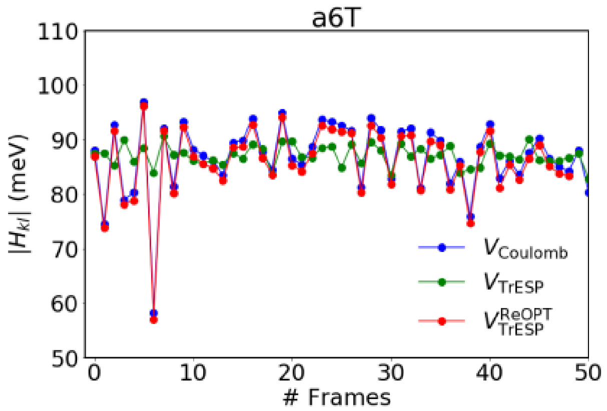

Supplementary Figure 4: TrESP approximations to the Coulomb coupling for a6T. Excitonic couplings obtained with frozen TrESP charges (fitted to different snapshots taken from an MD simulation) are shown in green. Excitonic couplings obtained with TrESP charges that were re-optimized at every molecular configuration/frame are shown in red ($V_{\text{TrESP}}^{\text{ReOPT}}$). Reference Coulomb excitonic couplings are shown in blue.

using electronic structure) are small compared to the actual mean coupling in a6T (i.e. only about 5% compared to the excitonic coupling). Thus, even though the fixed transition charge approximation cannot completely capture their magnitude, this is not expected to make any significant difference for the calculation of transport properties. Importantly, if the TrESP charges are updated every MD time step, $V_{\text{TrESP}}^{\text{ReOPT}}$, an almost perfect agreement with V_{Coulomb} is obtained, see Supplementary Figure 4.

We conclude by remarking that, despite the TrESP approximation inevitably leads to a small loss of accuracy when compared with the rigorous Coulomb integral calculation, especially for flexible molecules (e.g. polymers), it provides a very efficient scheme to evaluate a large number of coupling matrix elements along MD dynamics. It is very accurate when it comes to mean couplings and fluctuations of rigid conjugated molecules. Updating the transition charges along the MD is expected to improve the accuracy of TrESP charges for more flexible molecules. This could be achieved, for example, using interpolation schemes or machine-learning techniques[33].

Distance dependence of excitonic couplings

In order to explore the distance dependence and the long-range nature of the excitonic couplings, and to find how well the observations made before for the closest crystal pairs can be generalized to molecules further apart, we performed coupling calculations displacing the the two molecules forming the π-stacked pairs (P_b, of ANT and a6T and P_a, P_{a1} of PDI, DCVSN5, respectively) at various distances. The results are reported in Supplementary Figure 5 for some of the systems analysed in this work.

As expected, V, V_{Coulomb}, V_{PDA} and V_{TrESP}, all perform very similarly at large distances, that is when the higher multiple terms of the transition densities can indeed be neglected. We note that the total

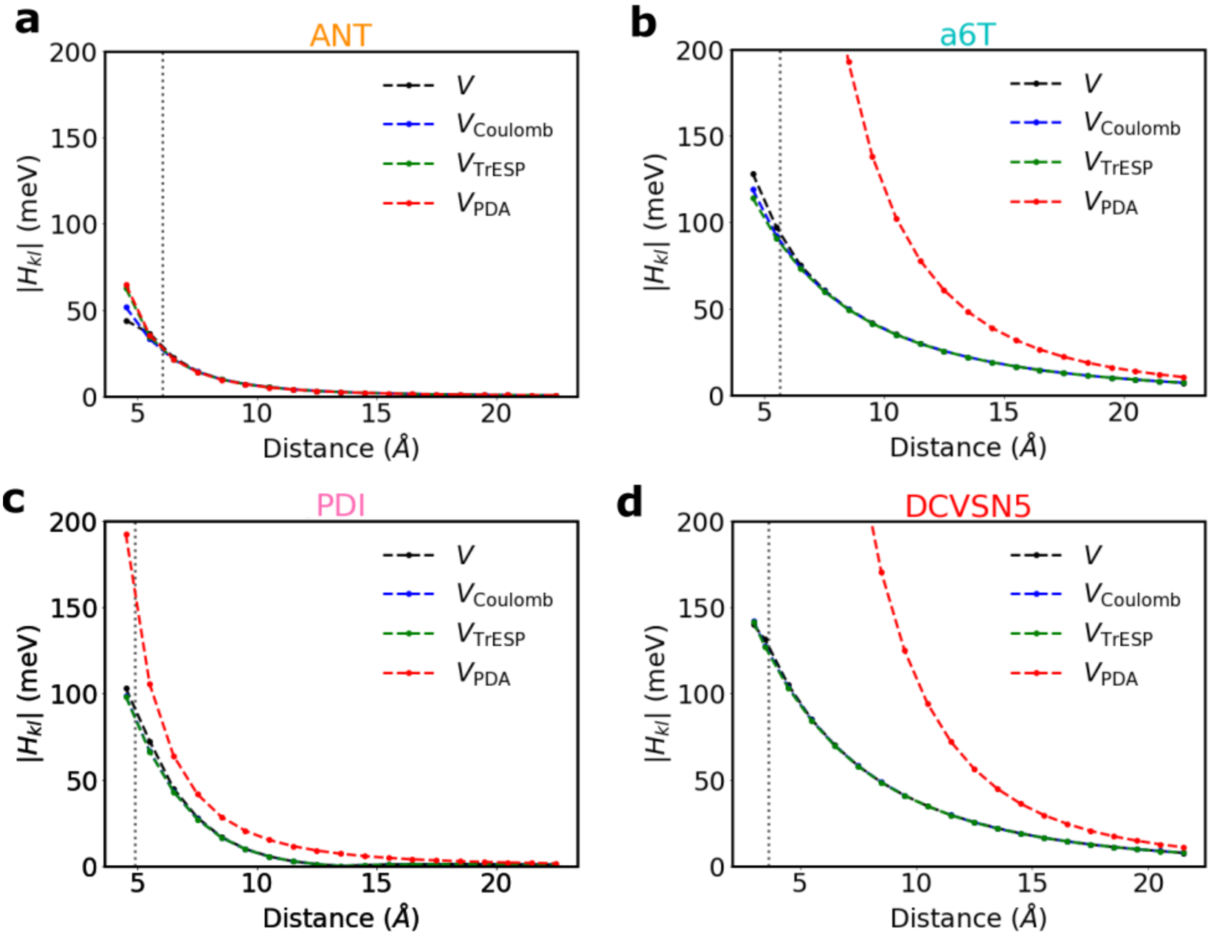

Supplementary Figure 5: Excitonic couplings as a function of distance for the pairs of ANT, a6T, PTCDI-H and DCVSN5 mentioned in the text. Couplings are given in absolute values and have been evaluated using MS-FED-FCD diabatization approach (using the first 20 excited states for the diabatization procedure), V_{Coulomb} (Eq. 11 main text), V_{TrESP} (Eq. 12 main text) and V_{PDA} (Eq. 8). The PDA clearly fails to provide an accurate description of the excitonic coupling at short intermolecular distances.

excitonic coupling, V, becomes quite small beyond 20 Å in all systems. Notably, as the distance decreases, and the closest distance between molecules in the crystal is approached (vertical dashed black line), the V_{PDA} approximation (red line) diverges from the other solid lines leading to strongly overestimated couplings for all the systems, but ANT. The reason is that the PDI molecule and, in particular, a6T and DCVSN5 have spatially extended transition densities and, when the distance between the sites falls below the actual spatial extent of the electronic transition density, the PDA breaks down and it should no longer be used (i.e. the actual shape of the transition density needs to be taken into account). This suggests caution in the application of Förster theory when describing exciton dynamics for molecular semiconductors composed of medium-sized and large organic molecules. On the opposite, the V_{TrESP} (green line) gives very good coupling estimates within the full distance regime, proving again to be a

useful method. We also note that, for all the systems investigated here, short range effects, V_{Short}, remain negligible even below the shortest crystal pair distance (dashed black vertical line), and the V_{Coulomb} is still a reasonably good approximation of the total V. Yet, short range effects might become important at even shorter distances when the overlap between donor acceptor wavefunctions becomes larger.

Transition dipoles

In Supplementary Figure 6 we show the transition dipoles obtained using TrESP charges.

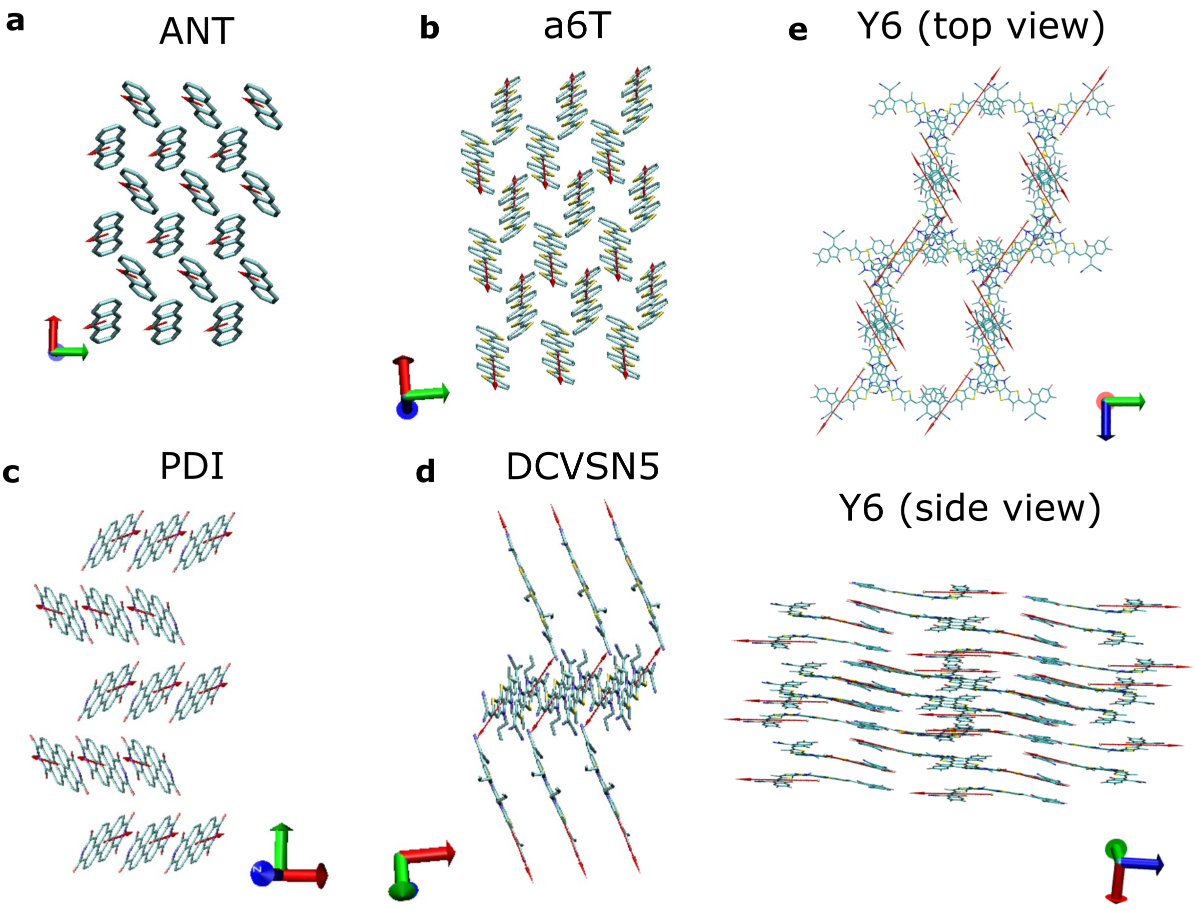

Supplementary Figure 6: Transition dipoles (obtained from TrESP charges) depicted on each molecule of the systems investigated.

Supplementary Note 4

FE-SH simulations

Force-field parametrization

The force field parameters for calculation of the site energies of the Hamiltonian Eq. 2 (in the main text), H_{kk}, are parametrized to reproduce the intramolecular exciton reorganization energy from TD-DFT calculations (as reported in Supplementary Table 2). In particular, the force field equilibrium bond lengths in the excited state, e.g., r_0^{ex}, are adjusted by adding scaled TDDFT displacements, $\Delta r_0^{\mathrm{TDDFT}}$, to the equilibrium bond lengths in the electronic ground state, r_0^{GS}, $r_0^{\text{ex}} = r_0^{\mathrm{GS}} + \beta \Delta r_0^{\mathrm{TDDFT}}$, until force field and TDDFT reorganization energies (Eq. 10 in the main text) match. If the scaling constant β is close to unity, the displacements in the force field and in TDDFT are almost identical. All other intra- and inter-molecular parameters were chosen to be the same as for the ground state. In Supplementary Figure 7, we report the bond length displacements in the S1 excited states relative to the ground electronic state and the β values for the different organic molecules considered in this work. As previously discussed, CAM-B3LYP functional was used for a6T, PDI and Y6, while ωB97X-D was employed for ANT and DCVSN5 for best comparison with Ref.[1]

The site energies H_{kk} of the Hamiltonian and their nuclear gradients $\nabla_I H_{kk}$ are obtained by assigning molecule k the force field parameters for the excited state and all other molecules $l \neq k$ the parameters for the ground state as previously done for the fragment orbital-based surface hopping (FOB-SH) in the context of charge transport.[34–36]. In the case of Y6, alkyl side-chains were removed from the simulation for simplicity and replaced by center of mass positional restraints of 1.255 kcal mol^{-1} Å^{-2} force constant to keep the structure close to the crystalline packing arrangement (RMSD 1.768 Å).

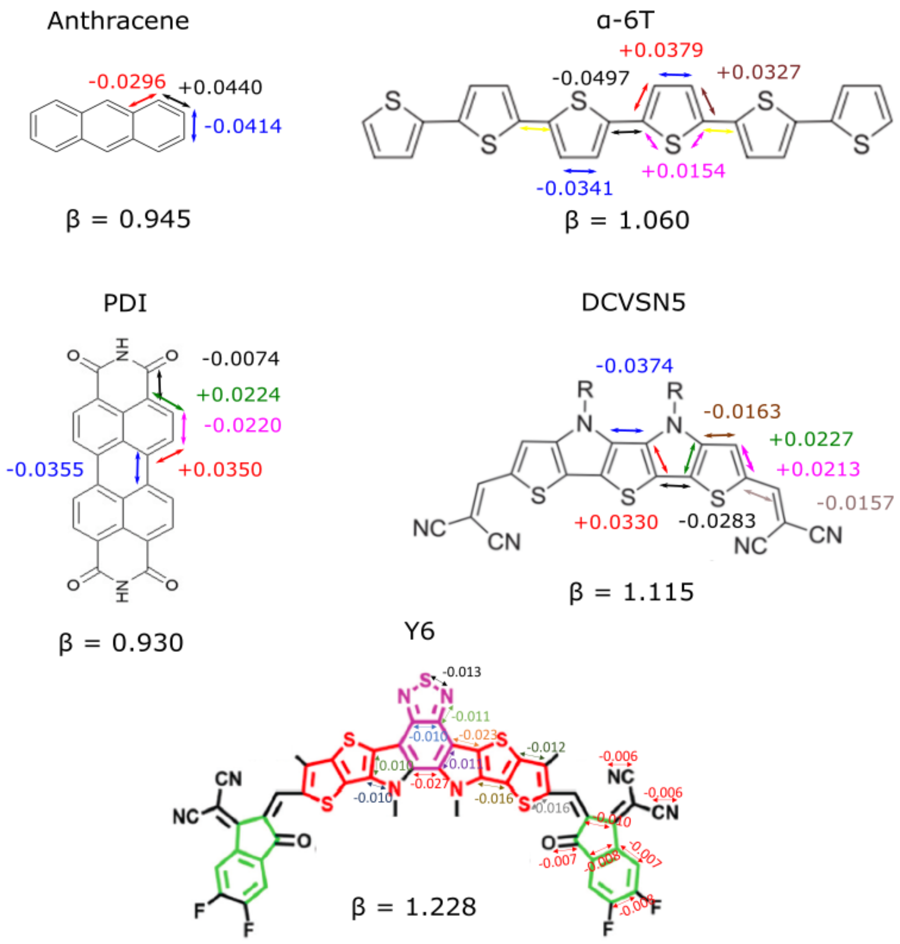

Supplementary Figure 7: Force field parametrization for singlet excitons. Changes in bond length upon change from ground to excitonic state, as obtained from TDDFT calculations. Displaced bond distances in Å are reported with different colours according to the displaced bonds. The + and − signs indicate an increase and decrease in bond length going from the ground to the excited system, respectively. The displacements are used to parametrize the force field for the molecules in their lowest energy singlet state. For clarity, displacements symmetric to the ones indicated are not shown. The scaling factor β for force field parametrization according to the TDDFT reorganization energy, as described in the text, is also reported for each system.

Cell parameters

Supplementary Table 4: Unit cell parameters (Å), transport direction investigated and related supercell information.

System	ANT	a6T	PDI	DCVSN5	Y6
a	8.562	9.140	4.865	7.341	14.469
b	6.038	5.684	14.660	27.685	21.186
c	11.184	20.672	10.844	12.527	30.842
α	90.000	90.000	90.000	90.000	109.352
β	124.700	97.780	91.330	103.828	96.264
γ	90.000	90.000	90.000	90.000	98.409
Z	2	2	2	4	4
Dir.	$a-b$	$a-b$	$a-b$	a	a
Super cells	12x16x2	16x16x2	24x12x2	54x2x2	48x2x1
Tot. atoms	18432	45056	46080	45792	32640

Mean squared displacements

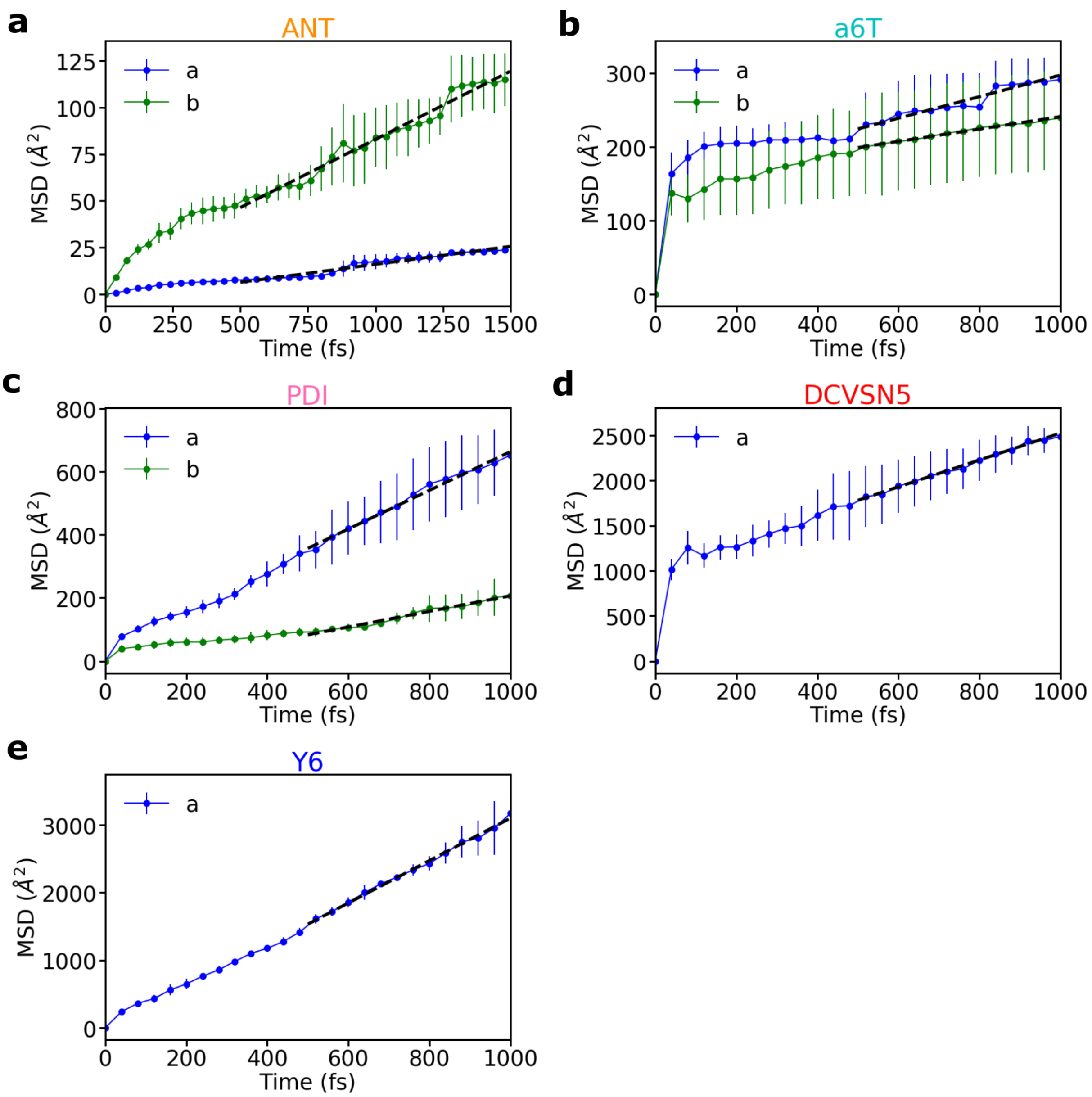

Supplementary Figure 8: Representative MSD for the investigated systems. The present simulations refer to 300 molecules of ANT, 312 of a6T, 336 of PDI, 300 of DCVSN5 and 360 of Y6. The MSDs along a and b crystallographic directions (blue and green, respectively) are obtained from the average of 600 FE-SH trajectories using Eq. 7 in the main text. For a6T and PDI about 450 trajectories were run. Error bars are obtained by block-averaging over 3 blocks, about 200 trajectories each. The diffusion constant is obtained from linear fits to the linear MSD portion (dashed black lines).

Convergence of exciton diffusion constants

Simulation details are given in *Methods* section of the main text.

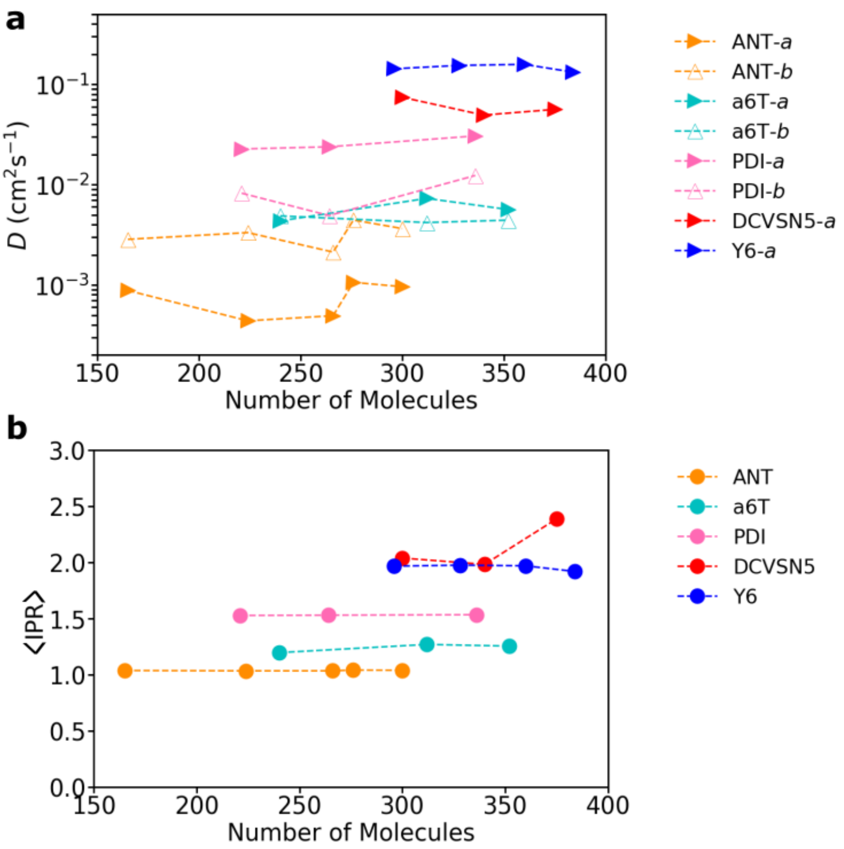

Supplementary Figure 9: Convergence Analysis (**a**) Convergence of the diffusion constant as a function of the number of electronically active molecules for the supercells used in this work. The diffusion constant is obtained from linear fits to the MSD after initial relaxation, as described in the main text. (**b**) Convergence of the average $\langle \mathrm{IPR} \rangle$ as a function of the number of active molecules. Super cells were elongated along the transport directions investigated. The time step used for FE-SH simulations was set to 0.01 fs for all systems in order to tackle as well as possible the trivial crossings issue.

Supplementary Note 5

Exciton transport analysis

Impact of isotropy and dimensionality on exciton transport

Using the transient localization theory framework[37,38] as well as direct non-adiabatic molecular dynamics simulations[35] in the context of charge transport in organic semiconductors, it was recently proven that charges diffuse more effectively in 2D (or 3D) systems featuring isotropic nearest neighbour electronic couplings, rather than in 1D-like materials with a sizeable electronic coupling along a particular transport direction. To asses the impact of dimensionality on the transport properties of excitons we performed exciton transport simulations on reduced 1D models. In particular, we focused our analysis on two extreme cases: ANT and Y6, displaying the lowest and the highest diffusion constants, respectively. For ANT we studied a 1D chain along b direction (see Figure 1 in the main text), while for Y6 a single pillar of molecules along a direction (out of the 4 pillars forming the rod-like nano-crystal adopted in this work) was activated to the transport of the exciton (see *Methods*). We found that in both cases the MSD and diffusion constant for reduced dimensionality systems are smaller compared to the respective larger models (see Supplementary Figure 10a,c). However the difference is marginally small for ANT (D_b(2D)$=3.7 \times 10^{-3}$ vs D_b(1D)$= 2.4 \times 10^{-3}$) and goes up to about factor 4 in Y6 (D_a(3D-like)$= 150 \times 10^{-3}$ vs D_b(1D)$= 39 \times 10^{-3}$). This can be readily explained by noticing that ANT shows a larger excitonic coupling along b direction compared to the other two crystallographic directions within the herringbone layer plane (see Table 1 in the main text), thereby making this system already 1D-like and quite strongly anisotropic for exciton transport (see also Figure 4a in the main text). On the other hand, Y6 shows couplings that are of the same order of magnitude between different molecular pairs owing to its 3D-like structure and favourable packing. Thus, in the latter case, artificially restricting exciton transport to take place along a single pillar of molecules makes the transport slower and less effective. The different coupling anisotropy characterizing the two systems is also reflected in the delocalization of the thermally accessible excitonic band states (see Supplementary Figure 10b,d). For ANT, these states have a similar delocalization for both 1D and 2D models. Whereas, for Y6 a larger model composed by multiple pillars consistently enhances the delocalization of the band edge states. This makes the transient delocalization mechanism more effective in the latter system.

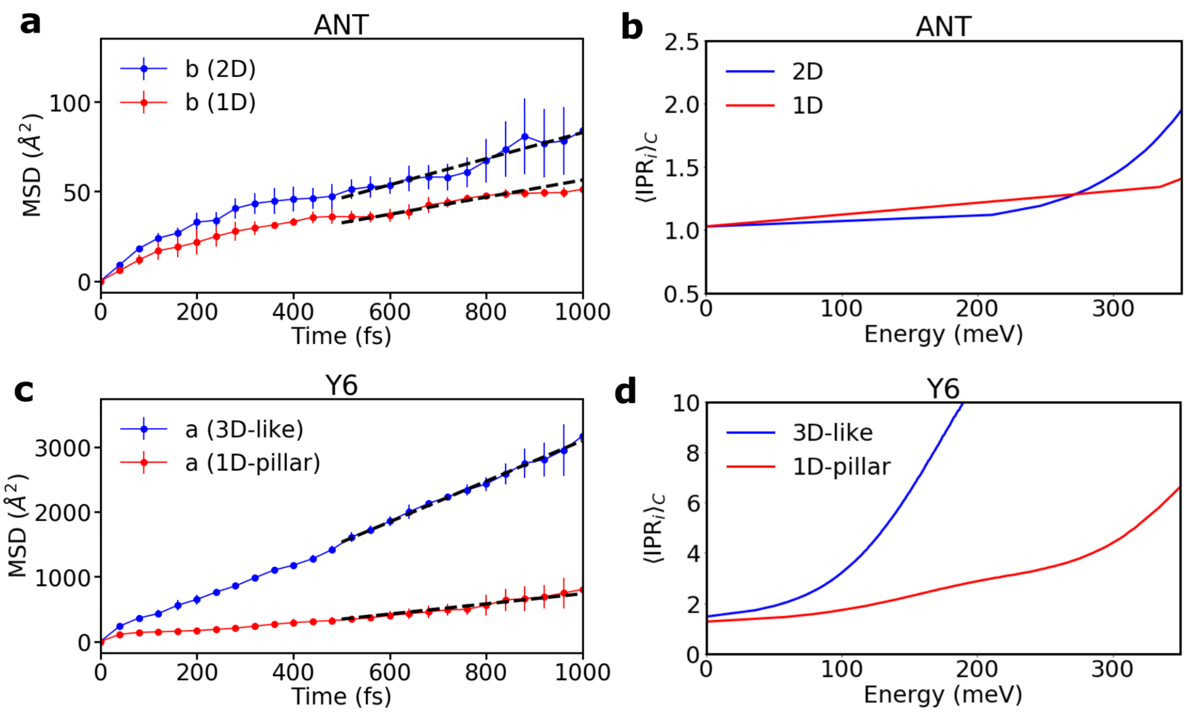

Supplementary Figure 10: Impact of dimensionality on the MSD and exciton states delocalization. Panels (**a**),(**c**) show the MSDs along the indicated crystallographic directions for ANT and Y6, respectively. Panels (**b**),(**d**) represent the $\langle \mathrm{IPR}_i \rangle_\mathrm{C}$ (Eq. 9 main text) of the adiabatic states (averaged over several trajectories and over snapshots) vs the energy of the states for the same systems. In all panels blue lines represent the results for larger supercell sizes, while red lines represent data for reduced dimensionality models as described in the text.

Impact of initial conditions on the diffusion

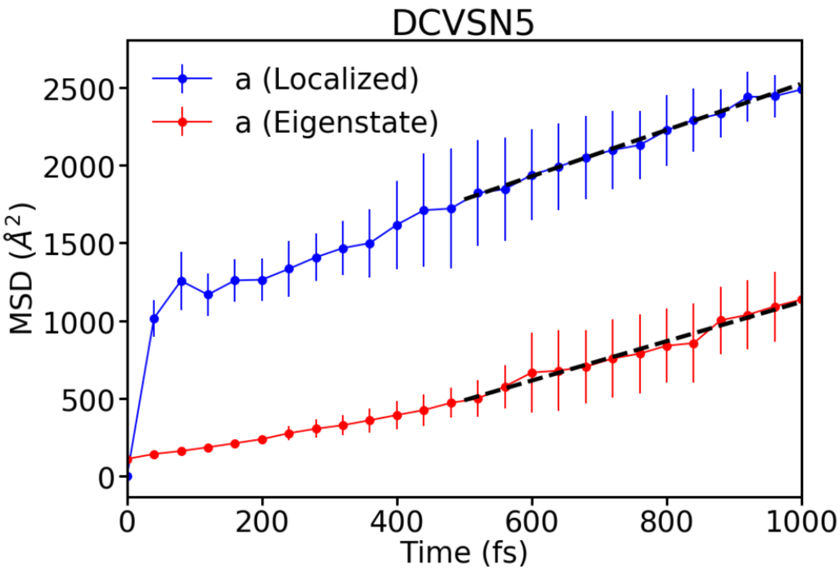

Supplementary Figure 11: Impact of initial conditions. The MSDs along a crystallographic direction of DCVSN5 were calculated starting with the charge fully localized on a given molecule (blue line), or starting from an eigenstate of the Hamiltonian at $t = 0$ (red line) located within $2k_BT$ from the bottom of the excitonic band of the system. Details for the two initial conditions are given in *Methods* of the main text. Notably, despite the different initial relaxation the diffusion constants (at long time) are the same for both starting conditions within the accuracy of the method.

Delocalization of thermally accessible states as a function of interactions range

It has been recently reported that the long-range excitonic interactions can drastically modify the exciton diffusion constant in ordered nanofibers of P3HT depending on the length of the polymeric fibers.[30,31] Prodhan and co-workers[31] found that the diffusion constant increases dramatically in long polymer chains when long-range interactions are included in the description. On the opposite, it was found that the same long-range interactions are responsible for a smaller value of the diffusion constant in shorter polymer chains (e.g. with 6 thiophene units).

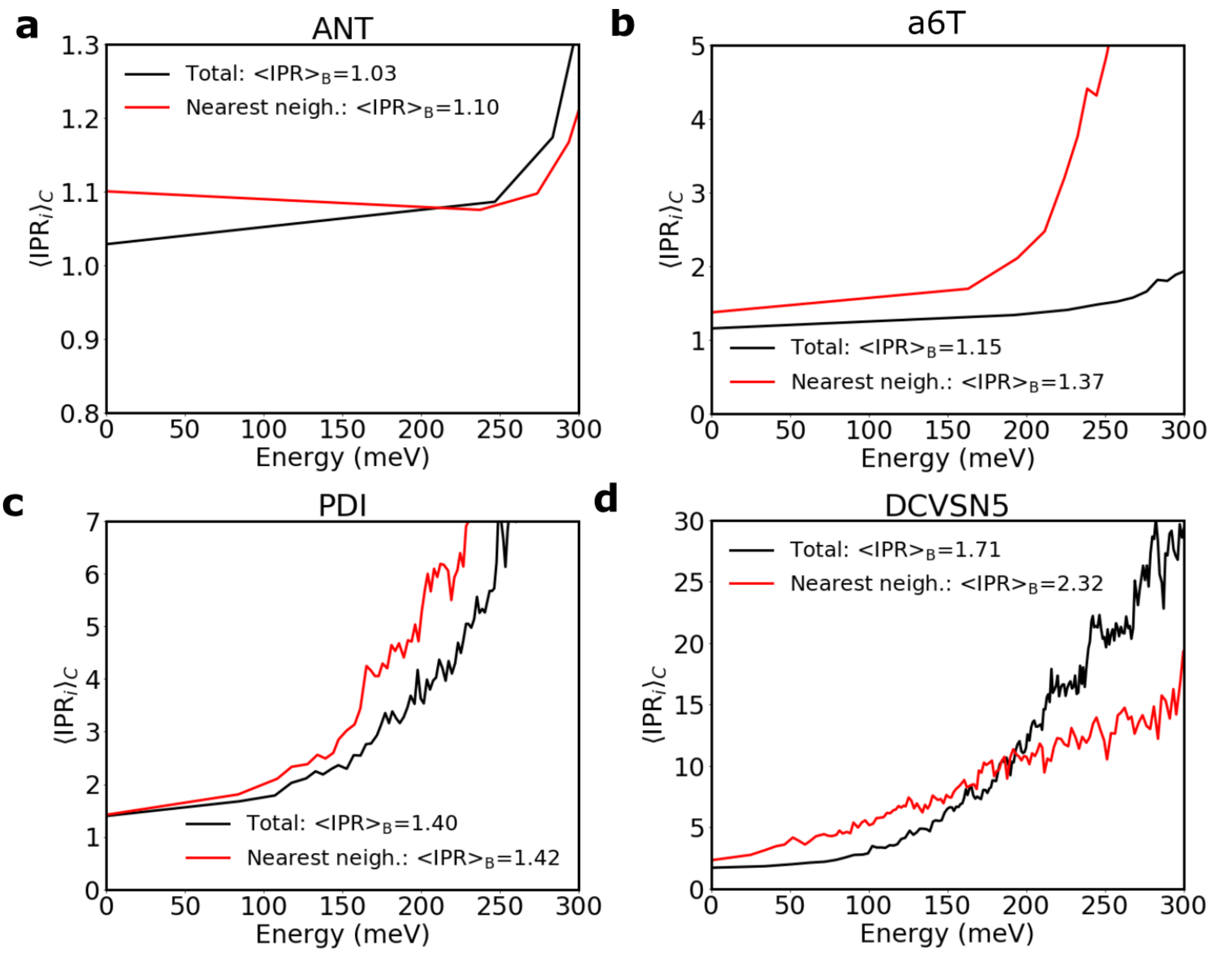

Supplementary Figure 12: Effect of removing long-range interactions on the delocalization of the states at the bottom of the excitonic band. The $\langle \mathrm{IPR}_i \rangle_\mathrm{C}$ (Eq. 9 main text) of the adiabatic states (averaged over snapshots of a single FE-SH trajectory) is plotted against their energies. In red we show simulations where only nearest neighbours are retained, while in black simulations where the full interaction range is considered. Note how the thermally accessible states below 200 meV are generally slightly more delocalized when only nearest neighbour interactions are considered.

In the present FE-SH simulations on the molecular semiconductors analysed in this work the full-range of interactions is considered in order to obtained the most accurate description possible for the

diffusion constant. It is interesting, however, to investigate the role of long-range interactions in the molecular systems presented here as a complement to the polymeric chains investigated in Ref.[30] and Ref.[31]. To estimate the impact that removing the long-range interactions has on the delocalization of the thermally accessible states of the excitonic band (and therefore on the diffusion constant), in Supplementary Figure 12 we plot the IPR of the states as a function of their energy (Eq. 9 main text), both when the full range of interactions is considered (black line) and when only nearest neighbours are retained (red line). Interestingly, we observe that in the latter case, the lower energy tail states becomes slightly more delocalized compared to the case where the full range of excitonic interactions is considered (note that this is no longer the case for the states in the middle of the excitonic band which are always more delocalized when long-range interactions are considered for the systems investigated). This increase in the extent of delocalization of the excited states can be quantified Boltzmann weighting the IPR_i (Eq. 9 main text), which for simplicity we indicate as $\langle\mathrm{IPR}\rangle_\mathrm{B}$. This value is larger when only nearest-neighbour interactions are retained in the Frenkel Hamiltonian. Thus, we expect long-range interaction to be indeed slightly detrimental for the exciton transport and diffusion constant in all these molecular systems (as it was found in short polymer chains[31]).

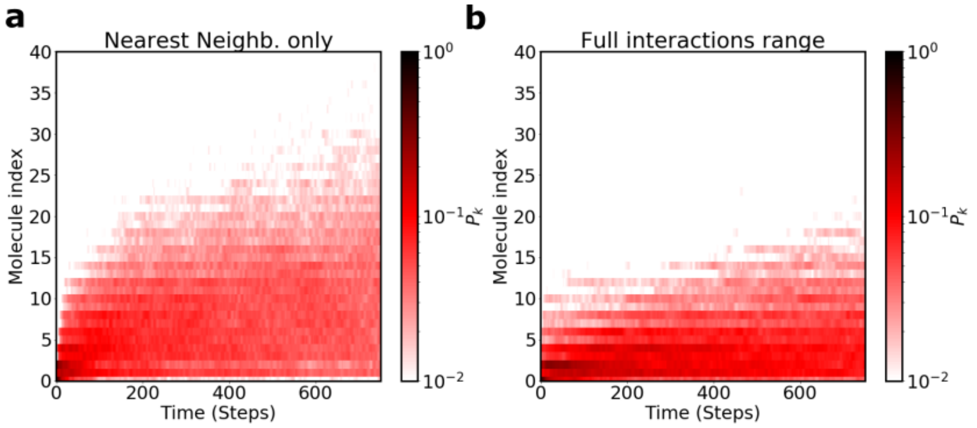

Supplementary Figure 13: Impact of long-range interactions on the wavefunction population of DCVSN5. Sum of the populations of 600 individual surface hopping simulations performed on 1D chain of DCVSN5. (**a**) Long-range exciton couplings were removed. (**b**) Long-range exciton couplings were retained. Note how the wavefunction populates many more molecules when long-range interactions are removed.

As additional verification of the (small) detrimental effect of long-range interactions on the exciton diffusion properties of these systems, we show in Supplementary Figure 13 that for a single chain of DCVSN5 the exciton extends over more sites when only nearest-neighbour interactions are considered. This observation acts as an additional (small) effect of why charge carrier (which are naturally subjected to short-range interactions) delocalize more than excitons in these molecular crystals.

Supplementary Note 6

Comparison of FE-SH diffusion constants with values from the computational and experimental literature

Anthracene

Anthracene represents a useful system to benchmark the FE-SH as the diffusion constant and related diffusion length have been evaluated by other authors using Marcus-like rate expressions[1] as well as Boltzmann-corrected Ehrenfest dynamics[39]. Some experimental estimates of the diffusion constant, that can be used as a suitable reference, are also available[40–42]. The reorganization energy of ANT is quite high compared to the excitonic coupling, supporting the formation of excitons mostly localized on a single molecule. The IPR calculated from FE-SH simulations, Eq. 8 (main text), is on average 1.04. This small IPR can be readily explained by considering that the barrier for exciton hopping between different molecules (estimated with Eq. 1) is about 112 meV which is much higher than the largest coupling in the crystal of about 30 meV and the thermal energy at room temperature (25 meV). This observation has justified the use of perturbative rate theories[1] to study the transition mechanism for this system (see below). Notably, as shown in Supplementary Figure 3 and already observed in the literature[1], the exciton couplings in anthracene exhibit sizeable thermal fluctuations compared to the mean coupling which provides a good motivation to explore direct dynamics methods (such as FE-SH and Ehrenfest) as well.

To assess the quality of FE-SH simulations we report in Supplementary Table 5 a comparison between the diffusion constants and related diffusion lengths (estimated using an exciton lifetime of $\tau = 10$ ns as found by experiments[40]) with the same values obtained from different computational methods and available experimental estimates. In particular, to calculate the diffusion constants, Elstner's group in Ref.[39] employed semi-empirical time-dependent density functional tight-binding (TD-DFTB) method in combination with Ehrenfest dynamics. The latter approach was opportunely corrected to approximately satisfy detailed balance (which is very important for an accurate dynamics as detailed in Ref.[31]). This approach is referred to as Boltzmann-corrected Ehrenfest method (BC-Ehrenfest). Using transition densities taken from TD-DFTB, the authors obtained averaged Coulomb couplings along MD ($\sqrt{\langle|H_{kl}|^2\rangle}$) of about 31 meV for P_b and 11 meV along T pairs. These values are very similar to what found in this work from the TrESP approach ($\sqrt{\langle|H_{kl}|^2\rangle}$ about 30 meV and 7 meV for P_b and T pairs, respectively). However a smaller reorganization energies of 302 meV was found in the former work, as a consequence of the approximate DFTB approach that relies on local functionals to describe the excited state geometry. This reorganization energy was employed to model the exciton-phonon coupling leading to a weaker ex-

citon relaxation compared to TDDFT used in FE-SH, where the exciton reorganization energy is 561 meV. Nevertheless, despite these differences and the different underlying dynamics, FE-SH and BC-Ehrenfest methods yield similar diffusion constants (see Supplementary Table 5). The diffusion lengths from FE-SH and BC-Ehrenfest are both slightly underestimated compared to the experiments (see main text for a discussion on this point).

Supplementary Table 5: Couplings (meV) for P_b pair, diffusion constants and diffusion lengths from different computational methods and experiments.[a]

	FE-SH[b]	BC-Ehrenfest[c]	MLJ-Rate[d]	Exp.[e]
$\sqrt{\langle \lvert H_{kl}\rvert^2\rangle}$ [f] (meV)	29.6	30.5	35.9	-
λ^{XT} (meV)	561	302 [g]	589 [h]	-
D_a (cm^2 s^{-1})	7.7E-04	7.1E-04	2.9E-03	1.8E-03
D_b (cm^2 s^{-1})	3.3E-03	2.4E-03	8.2E-03	5.0E-03
L_a (nm)	39	38	76	60
L_b (nm)	81	69	128	100

[a] The diffusion length is estimated as described in the main text for all the different methodologies using an exciton lifetime $\tau = 10$ ns, as obtained by experiments[40]. [b] FE-SH simulations are performed as described in *Methods*. [c] Boltzmann corrected Ehrenfest values are taken from Ref.[39]. The authors used reduced dimensionality 1D models along a and b crystallographic directions. [d] Perturbative rate theory result are taken from Ref.[1]. A rate expression very similar to the Marcus-Levich-Jortner rate for electron transfer was used by the authors by including an effective quantum mode coupled to the excitation energy transfer process. [e] Experimental estimated for the diffusion lengths are taken from Ref.[41,42]. [f] Excitonic couplings (averaged along MD trajectories) are given for P_b in the crystal. [g] Reorganization energy from TDDFTB [h] Reorganization energy obtained using normal mode analysis based on Huang-Rhys factors evaluation and the same level of theory as for FE-SH.

In Supplementary Table 5 we also compare both numerical non-adiabatic propagation schemes with the perturbative rate theory used in Ref.[1] by Aragó et. al as well. The latter approach is reasonably well justified in this system by the high local exciton-phonon coupling and the mostly incoherent hopping mechanism (observed by inspecting non-perturbative FE-SH trajectories, see e.g. Figure 3 main text). The rate expression used in Ref.[1] is very similar to the Marcus-Levich-Jortner rate for electron transfer[28,43]. It introduces an effective quantum high-frequency mode that is coupled to the exciton transfer process in order to effectively account for quantum-mechanical vibrations. In fact, in the common Marcus expression all vibrational modes in the system are treated as classical harmonic oscillators. While such an approximation is generally good for treating low frequency motions of a general solvent, it is not completely accurate for intra-molecular modes of the molecules, since for these modes $\hbar\omega > k_B T$. The approach employed by Aragó et al. gives a diffusion constant in the same order of magnitude as FE-SH and BC-Ehrenfest approaches and slightly longer diffusion length compared to both non-adiabatic propagation schemes. This can be explained with the fact that high frequency vibrations play a non-negligible role for the transport and, for systems with a reasonably high activation barrier (e.g. ANT), a classical treatment of modes might not be entirely justified (even at room temperature). This fact provides an

additional explanation for the lower diffusivity predicted by the FE-SH and BC-Ehrenfest approaches, which treat all the modes with classical force fields, compared to both experiment and Marcus-Levich-Jortner like expression. On the other hand, the excitonic couplings computed in Ref.[1] were found by using a diabatization scheme that, to the best of our understanding, includes only 2 adiabatic states[1]. Therefore the coupling between Frenkel exciton states is possibly an effective coupling still contaminated by mixing with charge-transfer states. For this reason, these couplings are slightly larger compared with both TrESP and TD-DFTB couplings (which by constructions do not include such a mixing with charge transfer states).

Overall, considering the differences in simulation set-ups, parameters entering the various Hamiltonians or analytic expressions and the uncertainty commonly characterizing experimental measurements, we show in Supplementary Table 5 that the agreement between FE-SH and other literature data is very encouraging.

a6T

The exciton diffusion length of a thin-film made of a6T molecules vacuum-deposited on quartz was reported to be about 60 nm by the quenching of the photoluminescence[44]. This value is close to the experimental diffusion length reported for anthracene, although for the latter system the exciton lifetime is an order of magnitude longer (10 ns) than that in a6T (1.8 ns)[45] and compensate for the smaller diffusion constant in ANT compared to a6T (see Table 2 in the main text). The diffusion length of a6T also compares quite nicely with our simulations. Indeed, FE-SH yields diffusion lengths of about $L_a = 46$ nm for this system, assuming that the transport occurs within the 2D herringbone layer of the crystal.

Despite the good agreement between experiments and FE-SH simulations, it is important to stress that the measured diffusion constant for a6T system does not refer to the crystal structure (studied herein), but it is rather characteristics of a thin-film of a6T molecules. Different structures and morphologies of the sample make difficult a like-for-like comparison between FE-SH simulations and experiments and this should be taken just as indicative.

PDI

In contrast to both a6T and ANT, PDI, despite having a larger diffusion constant (as found from FE-SH simulations) of about $D_a = 26 \times 10^{-3}$ cm^2 s^{-1}, shows a diffusion length of less than 1 nm due to the fast decay of the exciton to the ground state (of about 100 fs[46]). A negligible diffusion length was also measured in Ref.[46] for a thin-film PDI derivative with a linear butyl side-chain. However also in this case the comparison with FE-SH simulation can only be indicative because of the different chemical structure of the PDI molecule and the morphology characterizing experimental samples versus the crystalline

structure considered in our simulations.

DCVSN5

To the best of our knowledge, DCVSN5 experimental measurements for the diffusion length are not available. However this system has been simulated in Ref.[1]. By using Ehrenfest dynamics coupled to a model Hamiltonian supplemented with effective local and non-local exciton-phonon coupling interactions, Ref.[1] estimated an exciton diffusion constant of more than 1 cm^2 s^{-1} for DCVSN5, which is much higher than what we found in this work.[1] We identified several contributing factors that might have led to such a large diffusion constant; electronic decoherence in the Ehrenfest approach is missing which is likely to result in an overestimation of the population of more delocalized higher-lying excited states at longer timescales; excitonic couplings are overestimated if only two 2 adiabatic electronic states are included in the diabatization procedure, as shown in Supplementary Figure 2; neglect of excitonic couplings beyond nearest neighbours leads to slightly enhanced delocalized states at the bottom of the excitonic band, see Supplementary Figures 12 and Supplementary Figures 13 and related discussion.

Y6

Also in the case of Y6, the comparison between the experimental estimates of diffusion constant and diffusion length and FE-SH in Table 2 of the main text should be taken just as indicative. In fact, Y6 forms a thin-film in real devices. In order to measure the exciton diffusion length, authors in Ref.[16] measure the exciton-exciton annihilation rate γ and calculate the exciton diffusion constant D according to $\gamma = 4\pi D R_0$. The above equation can be derived through solving the 3D diffusion equation. In the deduction, the 3D dimensionality is embodied in the factor of 4π. In general the diffusion length is dependent on the system dimension and it can be defined $L = \sqrt{nD\tau}$. Where $n = 2, 4, 6$ for 1D, 2D and 3D diffusion respectively. Experimentally however, in Ref.[16], L is defined as $L = \sqrt{D\tau}$. Therefore in Table 2 of the main text we provided values from both definitions of the diffusion length applied to a 3D model.

Supplementary Note 7

Comparison exciton vs charge transport

In Supplementary Table 6 we compare transport parameters characterizing hole and exciton transport for some of the organic crystals investigated in this work. We report hole transfer interactions for the same crystal pairs in Figure 1 (main text) and hole reorganization energies and compared these with excitonic interactions (V) found using MS-FED-FCD and related reorganization energies.

The hole transfer couplings have been computed using two different approaches. On the one hand, we used the well-established fragment orbital density functional theory (FODFT) approach Ref.[34,35,47]. This method is related to the interaction between the diabatic state wavefunctions constructed from orbitals of the isolated donor and acceptor fragments (similarly to what happens in constraint density function theory (CDFT), but unlike in CDFT, the interaction between donor and acceptor fragments is neglected). FODFT calculations are carried out with the CPMD program package using the PBE exchange correlation functional as previously done in Ref[34,35,47]. We also used a projection operator-based diabatization (POD) approach as Ref.[48,49], as a further way of computing charge transfer couplings. This approach is a diabatization scheme based on the self-consistent DFT Kohn-Sham Hamiltonian of the donor-acceptor system in question, represented in an orthogonalised atomic orbital basis and partitioned into donor and acceptor blocks. Each block is separately diagonalised, defining unitary transformations which are used to transform the off-diagonal blocks. The resulting off-diagonal matrix elements may be identified as electronic couplings between the block-diagonal donor and acceptor states. The Kohn-Sham DFT calculations were carried out with the PBE functional, a DZVP basis set and GTH pseudopotentials on the neutral dimer (using CP2K program package). After block diagonalization, the Hamiltonian matrix element between the HOMO POD states on each molecule is taken as the electronic coupling matrix element. Both FODFT and POD couplings are scaled by a factor 1.3 (giving sFODFT and sPOD) for the best agreement with high-quality ab-initio coupling estimates as done in Ref.[47,48].

The first important observation is that, despite being very different approaches, FODFT and POD yield very similar coupling values for all the systems considered. For a given molecule, the largest hole transfer couplings are comparable in magnitude to the largest excitonic couplings (obtained with MS-FED-FCD as described in Supplementary Method 1. A major difference is that excitonic couplings tend to be more isotropic than hole transfer couplings (except in ANT). This is because the latter, depending on the nodal shape and overlap of neighbouring orbitals, are exponentially sensitive to changes in orientation, while this is less the case for the excitonic couplings which are dipolar interactions to first order. Another notable observation is that generally, the excitonic couplings fluctuations are smaller than the corresponding fluctuations of charge transfer couplings in the case of charge transport. It has been

Supplementary Table 6: Couplings (in absolute values) and reorganization energies for hole and exciton transport for the investigated systems. FODFT and POD couplings ware scaled by 1.3.[47,48] MS-FED-FCD couplings include 20 states in the diabatization procedure. Hole reorganization energies employ B3LYP/6-31G(d,p). All values in meV.

			Hole (meV)			Exciton (meV)	
		Dist (Å)	sFODFT	sPOD	λ^{h}	MS-FED-FCD	λ^{XT}
ANT	P_b	6.04	56.99	53.20	138	27.88	561
	T	5.24	25.22	23.93		6.40	
	P_a	8.56	0.16	0.28		4.35	
a6T	P_b	5.68	4.78	1.17	254	91.92	558
	T	5.38	47.35	46.39		101.15	
	P_a	9.14	0.21	0.23		42.01	
PDI	P_a	4.87	109.94	110.80	153	100.06	391
	T_1	9.47	0.38	0.42		31.97	
	T_2	9.40	0.95	0.89		17.89	
	T_3	11.60	1.61	1.39		14.01	
DCVSN5	P_{a1}	3.64	161.95	161.57	155	137.65	320
	P_{a2}	4.46	181.77	188.31		151.93	
	T_1	14.2	0.36	0.08		24.14	
	T_2	14.81	1.45	1.09		23.65	

pointed out before[35] that the coupling fluctuations actually enhance the delocalization of the states and help the transport in the hopping regime, while they become detrimental in the band-like regimes as they act as scattering sites. This observation give an additional explanation for the fact that exciton transport in the investigated systems (where there is a reasonably high barrier) is slower than the corresponding charge transport (see Figure 5 in the main text).

The most important difference between hole and exciton is the magnitude of charge reorganization energies as compared to the corresponding exciton reorganization energies. We note that, for comparison, hole reorganization energies were computed using B3LYP functional, which is the most common and widely used functional for electron/hole reorganization energies[3,14,50] because of its good agreement with UPS spectroscopic measurements.[51,52] Taking as an example the anthracene molecule (see Supplementary Figure 14), the hole reorganization energy is ≈ 140 meV while the relaxation of the exciton is about four times larger. This is due to the large charge redistribution occurring upon excitation. In particular, just by looking at the NTOs, it is evident that almost every bonding interaction in the HOMO becomes an anti-bonding interaction in the LUMO and vice versa. This causes each bond to become stronger (in case of a bonding interaction) or weaker (for an anti-bonding one) changing the length of the actual bond and causing a large structural modification upon excitation (see also Supplementary Figure 14). These sizeable reorganization energies, that in FE-SH are used to re-parametrize the classical FF as explained in the main text, are responsible for the strong exciton localization in these systems. Bond

lengths displacement used for the parametrization of the force field of the excitonic state are reported in Supplementary Figure 7.

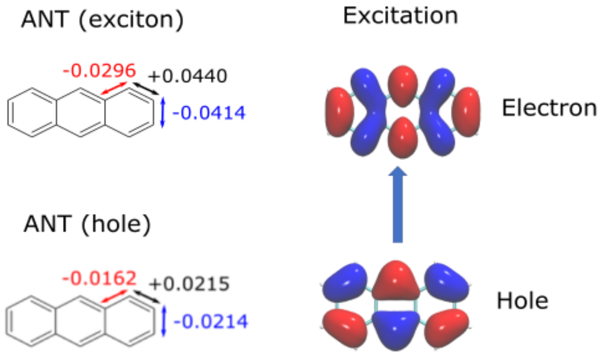

Supplementary Figure 14: Bond displacements for exciton and charge states. The + and − signs indicate an increase and decrease in bond length going from the ground (neutral) to the excited (charged) system for exciton (charge), respectively. The displacements are used to parametrize the force fields in FE-SH and FOB-SH respectively. Note that in this figure, bond displacements for ANT hole are taken from Ref.[34] and refer to B3LYP/6-311G(d) basis set. The NTOs related to the excitation of ANT are also shown.

Having previously analysed both charge and exciton transport parameters, we show in Supplementary Figure 15 a representative surface hopping trajectory for both transport cases within the herringbone plane of the anthracene crystal. The most striking difference is related to the transport mechanism of charge and energy carriers. Indeed, while the excitonic wavefunction is completely localized on a single molecule and it is mostly hopping from one molecule to a neighbouring one, the corresponding charge carrier wavefunction is much more delocalized ($\langle \mathrm{IPR} \rangle \approx 5$) and it is characterized by extended diffusive jumps as described in Ref.[35] for other high mobility organic crystal. The thermally accessible valence band states are more delocalized in ANT than the corresponding excitonic states, and this allows the charge carrier wavefunction to extend to several molecules (more than 15 in the example trajectory of Supplementary Figure 15) and to cover a longer spatial displacement compared to the localized exciton. This is a consequence of the more efficient transient delocalization. As we already showed in Figure 3 in the main text, a larger wavefunction delocalization correlates with a faster transport and a larger diffusion constant. In the particular case of ANT, the diffusion constant is much larger in the case of hole transport than to the corresponding exciton diffusion constant (D_b =0.0905 cm^2 s^{-1} vs 0.0033 cm^2 s^{-1}, respectively for similar system sizes).

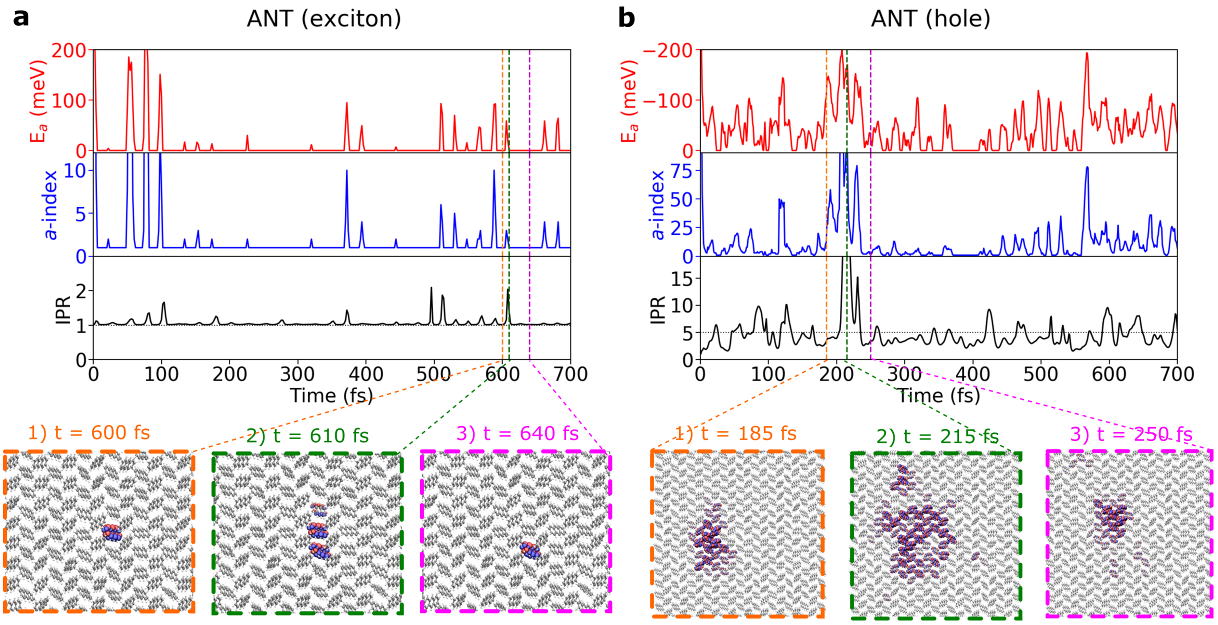

Supplementary Figure 15: Charge vs Exciton transport in ANT Hopping mechanism of exciton (**a**) and transient delocalization mechanism of electron hole transport (**b**) in ANT. See caption of Figure 3 main text for an explanation of the plotted quantities.

Supplementary References

[1] Aragó, J. & Troisi, A. Regimes of exciton transport in molecular crystals in the presence of dynamic disorder. *Adv. Funct. Mater.* **26**, 2316–2325 (2016).

[2] Yi, Y., Coropceanu, V. & Brédas, J.-L. A comparative theoretical study of exciton-dissociation and charge-recombination processes in oligothiophene/fullerene and oligothiophene/perylenediimide complexes for organic solar cells. *J. Mater. Chem* **21**, 1479 (2011).

[3] Carmen Ruiz Delgado, M., Kim, E. G., Da Silva Filho, D. A. & Bredas, J. L. Tuning the charge-transport parameters of perylene diimide single crystals via end and/or core functionalization: A density functional theory investigation. *J. Am. Chem. Soc.* **132**, 3375–3387 (2010).

[4] Landi, A. & Padula, D. Multiple charge separation pathways in new-generation non-fullerene acceptors: a computational study. *J. Mater. Chem. A* **9**, 24849–24856 (2021).

[5] Klevens, H. & Platt, J. Spectral resemblances of cata-condensed hydrocarbons. *J. Chem. Phys.* **17**, 470–481 (1949).

[6] Lambert, W., Felker, P., Syage, J. & Zewail, A. Jet spectroscopy of anthracene and deuterated anthracenes. *J. Chem. Phys.* **81**, 2195–2208 (1984).

[7] Janssen, R., Smilowitz, L., Sariciftci, N. & Moses, D. Triplet-state photoexcitations of oligothiophene films and solutions. *J. Chem. Phys.* **101**, 1787–1798 (1994).

[8] de Melo, J. S., Silva, L. M., Arnaut, L. G. & Becker, R. Singlet and triplet energies of α-oligothiophenes: A spectroscopic, theoretical, and photoacoustic study: Extrapolation to polythiophene. *J. Chem. Phys.* **111**, 5427–5433 (1999).

[9] Kircher, T. & Löhmannsröben, H.-G. Photoinduced charge recombination reactions of a perylene dye in acetonitrile. *Phys. Chem. Chem. Phys* **1**, 3987–3992 (1999).

[10] Mishra, A. *et al.* A-D-A-type S , N -Heteropentacenes: Next-Generation Molecular Donor Materials for Efficient Vacuum-Processed Organic Solar Cells. *Adv. Mater.* **26**, 7217–7223 (2014).

[11] Zou, X. *et al.* An Insight into the Excitation States of Small Molecular Semiconductor Y6. *Molecules* **25**, 4118 (2020).

[12] Martin, R. L. Natural transition orbitals. *J. Chem. Phys.* **118**, 4775–4777 (2003).

[13] Cupellini, L., Caprasecca, S., Guido, C. A. & Mennucci, B. NTOBuilder - A python tool to compute NTO orbitals and related metrics. (2020). URL `https://doi.org/10.5281/zenodo.3948078`.

[14] Stehr, V., Fink, R. F., Tafipolski, M., Deibel, C. & Engels, B. Comparison of different rate constant expressions for the prediction of charge and energy transport in oligoacenes. *Wiley Interdiscip. Rev. Comput. Mol. Sci.* **6**, 694–720 (2016).

[15] Arabi, S. A. *et al.* Nanoseed assisted PVT growth of ultrathin 2D pentacene molecular crystal directly onto SiO 2 substrate. *Cryst. Growth Des.* **16**, 2624–2630 (2016).

[16] Firdaus, Y. *et al.* Long-range exciton diffusion in molecular non-fullerene acceptors. *Nat. Commun.* **11**, 5220 (2020).

[17] Kupgan, G., Chen, X. K. & Brédas, J. L. Molecular packing of non-fullerene acceptors for organic solar cells: Distinctive local morphology in Y6 vs. ITIC derivatives. *Materials Today Advances* **11**, 0–7 (2021).

[18] Yanai, T., Tew, D. P. & Handy, N. C. A new hybrid exchange–correlation functional using the coulomb-attenuating method (cam-b3lyp). *Chem. Phys. letters* **393**, 51–57 (2004).

[19] Improta, R., Ferrer, F. J. A., Stendardo, E. & Santoro, F. Quantum-Classical Calculation of the Absorption and Emission Spectral Shapes of Oligothiophenes at Low and Room Temperature by First-Principle Calculations. *Chem. Phys. Chem.* **15**, 3320–3333 (2014).

[20] Chai, J.-D. & Head-Gordon, M. Long-range corrected hybrid density functionals with damped atom–atom dispersion corrections. *Phys. Chem. Chem. Phys* **10**, 6615–6620 (2008).

[21] Voityuk, A. A. & Rösch, N. Fragment charge difference method for estimating donor-acceptor electronic coupling: Applicati on to dna pi-stacks. *J. Chem. Phys.* **117**, 5607–5616 (2002).

[22] Yang, C.-H. & Hsu, C.-P. A multi-state fragment charge difference approach for diabatic states in electron transfer: Extension and automation. *J. Chem. Phys.* **139**, 154104 (2013).

[23] Hsu, C.-P., You, Z.-Q. & Chen, H.-C. Characterization of the Short-Range Couplings in Excitation Energy Transfer. *J. Phys. Chem. C* **112**, 1204–1212 (2008).

[24] Nottoli, M. *et al.* The role of charge-transfer states in the spectral tuning of antenna complexes of purple bacteria. *Photosynth. Res.* **137**, 215–226 (2018).

[25] Cupellini, L., Corbella, M., Mennucci, B. & Curutchet, C. Electronic energy transfer in biomacromolecules. *Wiley Interdiscip. Rev. Comput. Mol. Sci.* **9**, 1–23 (2019).

[26] Tölle, J., Cupellini, L., Mennucci, B. & Neugebauer, J. Electronic couplings for photo-induced processes from subsystem time-dependent density-functional theory: The role of the diabatization. *J. Chem. Phys.* **153**, 184113 (2020).

[27] You, Z.-Q. & Hsu, C.-P. Theory and calculation for the electronic coupling in excitation energy transfer. *Int. J. Quantum Chem* **114**, 102–115 (2014).

[28] Cupellini, L., Giannini, S. & Mennucci, B. Electron and excitation energy transfers in covalently linked donor–acceptor dyads: mechanisms and dynamics revealed using quantum chemistry. *Phys. Chem. Chem. Phys* **20**, 395–403 (2018).

[29] Azarias, C., Cupellini, L., Belhboub, A., Mennucci, B. & Jacquemin, D. Modelling excitation energy transfer in covalently linked molecular dyads containing a bodipy unit and a macrocycle. *Physical Chemistry Chemical Physics* **20**, 1993–2008 (2018).

[30] Sneyd, A. J. *et al.* Efficient energy transport in an organic semiconductor mediated by transient exciton delocalization. *Sci. Adv.* **7**, eabh4232 (2021).

[31] Prodhan, S., Giannini, S., Wang, L. & Beljonne, D. Long-range interactions boost singlet exciton diffusion in nanofibers of π-extended polymer chains. *J. Phys. Chem. Let.* **12**, 8188–8193 (2021).

[32] Huix-Rotllant, M., Tamura, H. & Burghardt, I. Concurrent effects of delocalization and internal conversion tune charge separation at regioregular polythiophene-fullerene heterojunctions. *J. Phys. Chem. Lett.* **6**, 1702–1708 (2015).

[33] Farahvash, A., Lee, C.-K., Sun, Q., Shi, L. & Willard, A. P. Machine learning frenkel hamiltonian parameters to accelerate simulations of exciton dynamics. *J. Chem. Phys.* **153**, 074111 (2020).

[34] Giannini, S. *et al.* Quantum localization and delocalization of charge carriers in organic semiconducting crystals. *Nat. Comm.* **10**, 3843 (2019).

[35] Giannini, S., Ziogos, O. G., Carof, A., Ellis, M. & Blumberger, J. Flickering Polarons Extending over Ten Nanometres Mediate Charge Transport in High-Mobility Organic Crystals. *Adv. Theory Simul.* **3**, 2000093 (2020).

[36] Ziogos, O. G., Giannini, S., Ellis, M. & Blumberger, J. Identifying high-mobility tetracene derivatives using a non-adiabatic molecular dynamics approach. *J. Mater. Chem. C* **8**, 1054–1064 (2020).

[37] Fratini, S., Ciuchi, S., Mayou, D., Laissardiere, G. T. d. & Troisi, A. A map of high-mobility molecular semiconductors. *Nat. Mater.* **16**, 998–1002 (2017).

[38] Fratini, S., Nikolka, M., Salleo, A., Schweicher, G. & Sirringhaus, H. Charge transport in high-mobility conjugated polymers and molecular semiconductors. *Nat. Mater.* **19**, 491–502 (2020).

[39] Kranz, J. J. & Elstner, M. Simulation of Singlet Exciton Diffusion in Bulk Organic Materials. *J. Chem. Theory Comput.* **12**, 4209–4221 (2016).

[40] Powell, R. C. & Soos, Z. G. Singlet exciton energy transfer in organic solids. *J. Lumin.* **11**, 1–45 (1975).

[41] Mulder, B. Anisotropy of light absorption and exciton diffusion in anthracene crystals determined from externally sensitized fluorescence. *Philips Res. Rep* **22**, 142–149 (1967).

[42] Mulder, B. Symmetry of the fluorescence and absorption spectra of anthracene crystals. *J. Phys. Chem. Solids* **29**, 182–184 (1968).

[43] Blumberger, J. Recent advances in the theory and molecular simulation of biological electron transfer reactions. *Chem. Rev.* **115**, 11191–11238 (2015).

[44] Mani, A., Schoonman, J. & Goossens, A. Photoluminescence Study of Sexithiophene Thin Films. *J. Phys. Chem. B* **109**, 4829–4836 (2005).

[45] Horowitz, G., Valat, P., Garnier, F., Kouki, F. & Wintgens, V. Photoinduced spontaneous and stimulated emission in sexithiophene single crystals. *Opt. Mater.* **9**, 46–52 (1998).

[46] Pandya, R. *et al.* Femtosecond Transient Absorption Microscopy of Singlet Exciton Motion in Side-Chain Engineered Perylene-Diimide Thin Films. *J. Phys. Chem. A* **124**, 2721–2730 (2020).

[47] Kubas, A. *et al.* Electronic couplings for molecular charge transfer: benchmarking cdft, fodft and fodftb against high-level ab initio calculations. *J. Chem. Phys.* **140**, 104105–21 (2014).

[48] Futera, Z. & Blumberger, J. Electronic couplings for charge transfer across molecule/metal and molecule/semiconductor interfaces: performance of the projector operator-based diabatization approach. *J. Phys. Chem. C* **121**, 19677–19689 (2017).

[49] Ziogos, O. G. *et al.* Hab79: A new molecular dataset for benchmarking dft and dftb electronic couplings against high-level ab-initio calculations. *J. Chem. Phys.* (2021).

[50] Yavuz, I., Martin, B. N., Park, J. & Houk, K. N. Theoretical study of the molecular ordering, paracrystallinity, and charge mobilities of oligomers in different crystalline phases. *J. Am. Chem. Soc.* **137**, 2856–2866 (2015).

[51] Malagoli, M., Coropceanu, V., Da Silva Filho, D. A. & Brédas, J. L. A multimode analysis of the gas-phase photoelectron spectra in oligoacenes. *J. Chem. Phys.* **120**, 7490–7496 (2004).

[52] Coropceanu, V. *et al.* Charge transport in organic semiconductors. *Chem. Rev.* **107**, 926–952 (2007).
